# Free and glycosylated aroma compounds in grapes monitored by solid‐liquid extraction and dispersive liquid‐liquid microextraction combined with gas chromatography‐mass spectrometry

**DOI:** 10.1002/jssc.202200181

**Published:** 2022-06-25

**Authors:** Ainhoa Oller‐Ruiz, Pilar Viñas, Manuel Hernández‐Córdoba, José Fenoll, Isabel Garrido, Natalia Campillo

**Affiliations:** ^1^ Department of Analytical Chemistry, Faculty of Chemistry, Regional Campus of International Excellence “Campus Mare Nostrum” University of Murcia Murcia Spain; ^2^ Sustainability and Quality Group of Fruit and Vegetable Products Murcia Institute of Agri‐Food Research and Development. C/ Mayor s/n. La Alberca Murcia Spain

**Keywords:** aroma compounds, dispersive liquid‐liquid microextraction, gas chromatography‐mass spectrometry, glycosides, grape

## Abstract

Fifteen aroma compounds have been determined in their free and glycosylated forms in grapes using dispersive liquid‐liquid microextraction with gas chromatography‐mass spectrometry. The sample treatment includes a previous solid‐liquid extraction stage and subsequent parallel microextraction approaches to preconcentrate total aroma content and the free fraction. Thus, the extraction of the total content of analytes requires previous enzymatic hydrolysis of the bound forms. For preconcentration, chloroform (250 μl) and acetonitrile (1.5 ml) were added to 10 ml of the sample extract in the presence of 0.5 g sodium chloride. The absence of matrix effect in the samples allowed quantification against aqueous external standards. Limits of detection ranged between 5 and 30 ng/g, depending on the compound. Method accuracy was studied through recovery assays, with recoveries in the 82–115% range being obtained. Relative standard deviations for repeatability studies were lower than 12%. Four different samples of grapes were analyzed, being quantified linalool in its free form at concentrations in the 359–470 ng/g range, and benzyl alcohol, 2‐phenylethanol, and linalool oxide I and II in their bound forms between 52 and 464 ng/g.

**Article Related Abbreviations**
DLLME: dispersive liquid‐liquid microextraction
HS: headspace
IS: internal standard
SLE: solid‐liquid extraction

## 1 INTRODUCTION

Organoleptic characteristics of fruit, and specifically of grapes, are related to their content in free volatile aroma compounds and to a lower extent to non‐volatile forms chemically bound to sugars. These glycosylated compounds can be hydrolyzed when grape turns into wine or by application of external enzymatic hydrolysis. The compounds responsible for the grape aroma are monoterpenes, sesquiterpenes, norisoprenoids, shikimic acid derivatives, benzene derivatives, aliphatic alcohols, sulfur compounds, carbonyl compounds, acids, esters, and lactones [1]. The relation between the chemical composition of grape and the variety to which it belongs has been widely studied [1, 2, 3]. For example, the highly appreciated floral aroma of the Muscat grape has been mainly attributed to monoterpenes, for which recent studies have demonstrated pharmacological properties [4].

The determination of aroma compounds has attracted the attention of researchers as regards grape origin, as well as its maturity stage, variety, or its adequacy as a table grape or for wine production. The literature reports several analytical methods for the determination of aroma chemicals in grapes [4, 5, 6, 7, 8, 9, 10, 11, 12, 13, 14, 15, 16, 17, 18, 19, 20, 21, 22, 23], both under their free forms and the more abundant glycosylate structures, in the latter case applying different glycolysis procedures, such as enzymatic and acid hydrolysis [5].

Because aroma compounds are usually found in grapes at trace levels, different preconcentration procedures have been applied to increase method sensitivity, in many cases also provide the cleaning of the sample matrix. SPE has been widely used for this purpose, allowing the isolation of the free and bound aroma fractions as well [4, 6, 8–11, 16, 19], with the disadvantages associated with conventional extraction methods, such as the high organic solvents consumption as well as the long analysis times required. Microextraction techniques have overcome many of these disadvantages, being applied under different modalities for preconcentration in wines and grapes [24]. Headspace SPME (HS‐SPME) [2, 13–15, 20, 25–29] has been by far the miniaturized technique more widely selected, in some instances in combination with SPE [7, 12, 19], and their features being recently compared [30]. In fact, HS‐SPME has also provided very good results for preconcentrating aroma compounds from avocado [31], apples [32], and ornamental plants [33, 34]. Other microextraction techniques such as stir bar sorptive extraction [17, 21, 22, 35], HS solid‐phase dynamic extraction [36], and dispersive liquid‐liquid microextraction (DLLME) [37, 38] have also been applied. On the other hand, HS sorptive extraction [39], micro‐matrix solid‐phase dispersion [40], and single drop microextraction [41] have also demonstrated their efficiency for preconcentrating aroma compounds from other sample matrices different from wines and grapes.

Considering the high volatility of aroma compounds, the analysis of the sample extracts is generally carried out by GC coupled with MS [2, 4, 6–17, 19–22, 25–32, 34–36, 42] or flame ionization detector [43]; although LC [37, 41, 44] and TLC [45] have also been applied.

In this paper, a procedure based on DLLME is developed for the preconcentration of fifteen aroma compounds, including thirteen monoterpenes, under their free and bound fractions, and two aromatic alcohols in the *Vitis vinifera* grapes, using GC‐MS for the analysis of the obtained extracts. The novelty of the study lies in the fact that it is the first time that the determination of volatile aroma compounds in grapes, both under their free and glycosylated forms, has been carried out using DLLME, minimizing in this way the use of organic solvents for preconcentration purposes.

## 2 MATERIALS AND METHODS

### 2.1 Chemicals

High‐quality ethanol, ACN, and chloroform were purchased from Sigma‐Aldrich (St. Louis, MO, USA). Analytical standards of α‐terpineol, nerol, geraniol, and eugenol, as well as linalool oxide (mixture of I and II isomers, 97% purity), rose oxide (mixture of I and II isomers, 98%), with the specified purities, were obtained from Fluka (Buchs, Switzerland), limonene (analytical standard) from Sigma‐Aldrich, and myrcene (90%), benzyl alcohol (99.5%), linalool (97%), 2‐phenylethanol (> 99%), citronellol (95%) and citral (95%) from Acros Organics (Geel, Belgium). Thymol (Acros Organics, extra pure) was used as an internal standard (IS). The molecular structures of the terpenes studied appear in Figure S1. Individual stock standard solutions of 200 μg/ml were prepared in methanol and stored in amber vials at –20°C. Working mixed standard solutions (5 μg/ml) were daily prepared by diluting the concentrated solutions with pure water and stored in the fridge. A Milli‐Q system (Millipore, Bedford, MA, USA) was used to purify the used water. D‐Gluconic acid lactone, polyvinylpolypyrrolidone, trisodium citrate dihydrate, potassium dihydrogen phosphate, sodium chloride, and *ortho*‐phosphoric acid (85% purity), obtained from Sigma, was used in the sample treatment. The commercial enzymatic preparation AR‐2000 with specific glycosidase side activities was provided by Gist Brocades (Seclin, France).

### 2.2 Instruments

Chromatographic separation was carried out with an Agilent HP 6890 GC (Agilent, Waldbronn, Germany) system using an HP‐5MS UI (30 × 0.25 mm i.d., 0.25 μm, Agilent) capillary column and helium as a carrier gas at a constant pressure of 7 psi. The injector port was held at 250°C, and 2 μl‐volumes were injected in the splitless mode (1 min). The oven program consisted of a temperature increase from 60 to 140°C at 3°C/min, which provided the elution of the compounds with retention times in the 6–22 min range (Table 1). The run chromatographic analysis took 27 min.

**TABLE 1:** Retention times and monitored ions for the aromatic compounds

**Compound**	***t*_R_ (min)**	**T (*m/z*)**	**Q1 (*m/z*)**	**Q2 (*m/z*)**	**Q3 (*m/z*)**	**Q1/T**	**Q2/T**
Myrcene	6.51	41	93	69	91	95.2	77.0
Limonene	7.89	68	67	93	79	75.5	73.4
Benzyl alcohol	8.21	79	108	107	77	92.0	60.4
Linalool oxide II	9.34	59	94	93	68	45.9	37.2
Linalool oxide I	9.91	59	94	93	68	44.9	34.8
Linalool	10.4	71	93	80	67	78.1	29.5
Rose oxide II	10.8	139	69	83	67	51.9	26.7
2‐Phenylethanol	11.2	91	92	122	65	53.1	24.5
Rose oxide I	12.1	139	69	83	67	53.2	24.8
α‐Terpineol	14.0	59	93	121	136	68.8	52.7
Citronellol	15.8	69	67	82	68	45.8	29.3
Nerol	15.8	69	67	93	68	38.6	26.7
Geraniol	16.7	69	93	68	67	22.4	17.5
Citral	17.5	69	84	53	94	23.6	16.6
Thymol (IS)	18.5	135	150	115	136	30.3	17.2
Eugenol	21.2	164	77	103	149	43.9	39.8

Abbreviations: T, target ion; Q, qualifier ions; Q/T, qualifier to target abundance ratio.

An Agilent 5973N quadrupole mass spectrometer was coupled to the chromatograph and the ionization was carried out in the electron ionization mode, operating with 70 eV as ionizing energy. The temperatures of the ion source and the quadrupole were 230 and 150°C, respectively. The electron multiplier voltage was set at 1300 V. Selected ion monitoring mode, using one target and three qualifier ions (Table 1), was adopted for quantification purposes to improve method sensitivity. The retention time for the target ion and qualifier‐to‐target ratio abundances were used for the identification of each compound. Injection of individual standard solutions, under the same experimental conditions using full scan mode with *m/z* in the 40–600 range, was used to obtain target and qualifier abundances.

A Dangoumau ball grinder was used to crush the grape samples under liquid nitrogen. An Eppendorf centrifuge model 5810R (Hamburg, Germany) and an IKA orbital shaker (Staufen, Germany) were also used in the sample treatment.

### 2.3 Samples and analytical procedure

Four different types of grapes, whose characteristics are shown in Table 2, were experimentally grown in a vineyard at the Instituto Murciano de Investigación y Desarrollo Agrario y Alimentario (IMIDA, Murcia, Spain) and sampled during the 2017 vintage. Grape samples were maintained at –20°C until analysis.

**TABLE 2:** Characteristics of the grapes analysed

**Characteristics**	**Grape 1**	**Grape 2**	**Grape 3**	**Grape 4**
Colour	White	White	White	Red
Texture (Newtons)	Crunchy: 22–24	Crunchy: 21–24	Crunchy: 21–24	Crunchy: 20–23
Caliber (mm)	18–21	19–22	19–22	18–23
Fertility rate	0.8–1.2	0.8–1.1	1.0–1.2	1.6–1.8
Bunch weight (g)	450–600	500–650	500–650	350–500
Collection period	End of June–Mid September	MidJune–Mid October	Mid‐August–Mid‐October	End of July–Mid‐August

Sample treatment included a previous grinding step under liquid nitrogen of the unraveled fruit (200 g) using a ball grinder, a powdered sample being obtained to be submitted to a solid‐liquid extraction (SLE) based on a previously applied procedure [4]. For SLE, 30 ml of water and 75 mg of D‐gluconic acid lactone were added to 7.5 g of the powdered sample (containing the IS at 80 ng/g), acting the lactone as an inhibitor of β‐glucosidase activity, thus preventing the natural decomposition of glycosylated aroma compounds. The mixture was orbitally shaken for 15 min at 4°C, and centrifuged at 9000 g for 20 min, maintaining a constant temperature of 4°C. The supernatant was filtered twice with glass wool, and 150 mg of polyvinylpolypyrrolidone were added to the filtrate, in order to eliminate the high levels of phenolic compounds from the sample matrix, which could affect glycosidase activity, and the mixture was stirred for 15 min and centrifuged at 9000 g during 10 min at 4°C.

The quantification of the total content of aroma compounds implied an enzymatic hydrolysis step to be applied to 10 ml of the obtained filtrate [4, 8, 9], which consisted of adding 200 mg of the enzyme AR‐2000, 0.5882 g of trisodium citrate dihydrate and 0.2722 g of potassium dihydrogen phosphate, the pH is adjusted to 5 with *ortho*‐phosphoric acid. The mixture was kept for 16 h at 40°C in a water bath to let the hydrolysis go.

**FIGURE 1:**
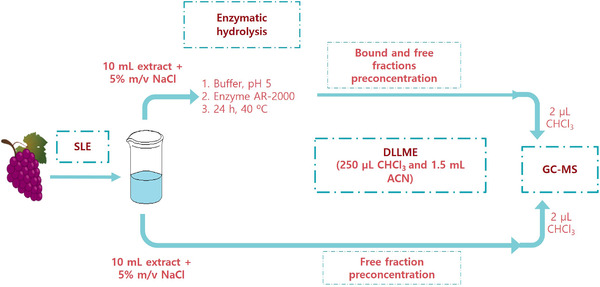
Scheme of the sample procedure applied

Preconcentration of the hydrolyzed and non‐hydrolyzed extracts by DLLME was carried out in parallel with the aim to distinguish between the aroma compounds content in both free and bound forms. Thus, 10 ml of the filtrate was placed in a conical‐bottomed glass centrifuge tube where 0.5 g NaCl had been previously weighed. A mixture composed of 1.5 ml of ACN (disperser solvent) and 250 μl of chloroform (extractant solvent) was quickly injected into the aqueous sample extract by means of a microsyringe, the turbidity produced by the dispersion of the extractant solvent being immediately observed. The ternary mixture was centrifuged at 3000 rpm and 4°C for 3 min and the sedimented organic phase was collected by means of a microsyringe. A volume of around 200 μl of enriched solvent was recovered, being a 2 μl‐aliquot directly injected into the GC‐MS system. All the samples were analyzed in triplicate. Figure 1 shows a scheme of the sample treatment.

Since no certified reference material was available for method validation, recovery studies were performed by fortifying two grape samples at two levels of concentration, 150 and 250 ng/g. For this purpose, once samples were ground, 225 and 375 μl of the 5 μg/ml aqueous working solution, as well as 120 μl of the 5 μg/ml IS solution, were added to 7.5 g of powdered sample, and the mixture was vigorously shaken and left to stand for 1 h at 4°C, before proceeding with the analysis following the previously described protocol.

## 3 RESULTS AND DISCUSSION

### 3.1 Optimization of the DLLME procedure

The optimal extractant and disperser solvents were selected using a 10‐ml volume of a sample extract obtained by SLE following the protocol of Fenoll et al. [4], and the supernatant was twice filtered through glass wool and fortified with the analytes at 50 ng/ml and the IS at 80 ng/ml. Other experimental conditions applied for these preliminary experiments were 300 μl of extractant solvent, 2 ml of disperser solvent, no salt was added to the aqueous phase, and emulsion disruption by centrifugation for 3 min at 3000 rpm and 4°C. Considering previous results proving higher extraction capability of organic solvents denser than water for monoterpenes preconcentration [37], chloroform, 1,2‐dichloroethane, dichloromethane, and carbon tetrachloride were assayed as DLLME extractants, using ACN as disperser solvent. Chloroform provided the highest extraction efficiency for all compounds, as resulted from the comparison of the peak areas obtained for each analyte divided by that of the IS, being, therefore, chloroform selected. When ethanol, ACN, methanol, and acetone were used as disperser solvents, turbidity was observed in the chloroform phase in all cases except when ACN was used for dispersing the extractant phase. Therefore, ACN was selected as disperser solvent with chloroform as extractant solvent.

A Taguchi orthogonal design was applied to study the influence on DLLME extraction efficiency of three factors (each one studied at three levels): volume of chloroform (250, 300, and 350 μl), the volume of ACN (0.5, 1.5, and 2.5 ml), and concentration of sodium chloride (0%, 5%, and 10% m/v) in the aqueous phase. The effects of the factors on the average extraction efficiency of the studied volatile analytes showed the maximum efficiency for the lowest extractant volume assayed, probably due to a dilution effect, whereas the best results for the other two variables were obtained with the central level tested (Figure S2). Consequently, the selected conditions corresponded to 250 μl of chloroform, 1.5 ml of ACN, and 5% w/v NaCl.

### 3.2 Enzymatic hydrolysis

As mentioned above, aroma compounds are found in grapes in significant proportions linked to the fruit's own sugars forming glycosides. For determination of the free forms, in addition to acid hydrolysis [6], the release of bounded compounds has also been carried out using pectinolytic enzyme preparations [7, 10], the commercial AR‐2000 enzyme [4, 8, 9, 11, 12], and glycosidases of vegetable or microbial origin, such as yeasts [14, 16]. Previous studies have shown a more specific bound aroma compounds hydrolysis capacity for the commercial enzyme AR2000, with a higher yield than other enzymes, such as those of microbial origin [12]. On the other hand, Schneider et al. observed that acid hydrolysis of terpenes produces a rearrangement of aromatic rings, giving rise to different compounds [8]. Considering these previous studies, the use of the commercial enzyme AR2000 was here adopted under slightly modified conditions than those applied by Fenoll et al. [4].

Separation by SPE of the free and bound forms has been previously proposed, to carry out the hydrolysis directly in the bound phase extract [4, 8, 9, 11, 12]. In the procedure here developed, the bound fraction was quantified by calculating the difference between the results obtained in the quantification of the total content, provided by the enzymatically digested sample extract, and the free fraction.

### 3.3 Validation of the method

To study the possible presence of the matrix effect, the standard additions method was applied to two grape samples, by analyzing two aliquots of unfortified sample and spiked aliquots at five concentration levels ranging between 25 and 400 ng/g, depending on the compound, and the IS was at 80 ng/g. The absence of thymol in the studied grape samples was previously checked in order to be used as IS.

The calibration graphs were obtained by representing the ratio between the analyte peak area and the IS peak area versus analyte concentration, and the slopes obtained were compared with those found by calibration against aqueous standards (Table 3). The application of an analysis of variance test provided “*p*” values in the 0.05–0.30 range, depending on the compound, concluding that there were no significant differences between the slopes of aqueous calibration and those obtained through standard additions, for both enzymatically digested and non‐digested sample extracts. Therefore, the quantification of the samples was carried out against aqueous standards, thus avoiding the use of the laborious method of standard additions.

**TABLE 3:** Slopes^a^ for aqueous calibration and standard additions for two samples (g/μg)

**Compound**	**Aqueous calibration**	**Grape 1**	**Grape 4**
Myrcene	2.9 ± 0.1	3.1 ± 0.2	2.9 ± 0.1
Limonene	3.8 ± 0.1	3.7 ± 0.1	3.8 ± 0.1
Benzyl alcohol	2.2 ± 0.1	2.1 ± 0.1	2.3 ± 0.2
Linalool oxide II	1.5 ± 0.1	1.5 ± 0.1	1.5 ± 0.1
Linalool oxide I	1.5 ± 0.1	1.5 ± 0.1	1.6 ± 0.1
Linalool	4.3 ± 0.3	4.2 ± 0.1	3.8 ± 0.3
Rose oxide II	1.7 ± 0.1	1.6 ± 0.1	1.7 ± 0.1
2‐Phenylethanol	4.7 ± 0.2	4.6 ± 0.1	4.5 ± 0.3
Rose oxide I	3.9 ± 0.1	3.8 ± 0.1	4.0 ± 0.2
α‐Terpineol	2.5 ± 0.1	2.5 ± 0.1	2.6 ± 0.1
Citronellol	1.5 ± 0.1	1.4 ± 0.1	1.5 ± 0.1
Nerol	1.3 ± 0.1	1.2 ± 0.1	1.3 ± 0.1
Geraniol	5.9 ± 0.1	5.8 ± 0.1	5.7 ± 0.1
Citral	3.5 ± 0.1	3.4 ± 0.1	3.4 ± 0.1
Eugenol	3.4 ± 0.1	3.4 ± 0.1	3.5 ± 0.1

^a^ Mean value ± standard error (*n *= 7).

The LODs and LOQs were calculated for S/N of 3 and 10, respectively, provided by chromatograms obtained from the SLE‐DLLME with GC‐MS of fortified sample matrices. As shown in Table 4, the LODs were between 5 and 30 ng/g (Table 4). Thus, the DLLME preconcentration procedure allowed a high grade of sensitivity to be obtained for both fractions in a compatible solvent for GC analysis. Enrichment factors were calculated by comparing the slopes obtained for calibration graphs obtained in the presence and in the absence of the DLLME step. Enrichment factor (EF) values were in the 25–36 range depending on the compound (Table 4).

**TABLE 4:** Analytical characteristics of the solid‐liquid extraction–dispersive liquid‐liquid microextraction (SLE‐DLLME) with GC‐MS developed method

			**RSD** **^c^** **(%)**		
**Compound**	**LOD** **^a^** **(ng/g)**	**LOQ** **^b^** **(ng/g)**	**LOQ level**	**200 ng/g**	**EF**
Myrcene	30	100	9.2	8.4	27
Limonene	21	70	9.0	8.3	30
Benzyl alcohol	5	17	12	10	32
Linalool oxide II	15	50	8.3	3.5	28
Linalool oxide I	15	50	7.9	4.9	29
Linalool	14	47	6.8	2.9	27
Rose oxide II	19	62	9.3	3.0	29
2‐Phenylethanol	12	41	11	8.1	25
Rose oxide I	16	53	8.0	4.0	32
α‐Terpineol	25	82	7.7	3.2	34
Citronellol	30	100	6.3	3.7	30
Nerol	24	80	10	1.7	36
Geraniol	30	100	9.8	4.3	32
Citral	19	63	12	6.5	29
Eugenol	14	49	11	4.4	33

^a^ Calculated for S/N = 3.

^b^ Calculated for S/N = 10.

^c^ n = 10.

The consecutive analysis of 10 aliquots of a grape sample fortified with all the analytes at their corresponding LOQ levels and 200 ng/g allowed estimating the repeatability of the proposed SLE‐DLLME with GC‐MS method. The values of RSD were between 1.7 and 12%, which denotes a good precision of the method (Table 4).

The accuracy of the method was verified by means of recovery studies, by fortifying two samples at the concentration levels of 150 and 250 ng/g. Recovery rates were obtained by comparing the obtained analytical signal to IS ratios with those predicted by the corresponding regression lines and varied between 82 and 115% (Table S1), corresponding to 82–115 and 97–115% ranges for the lowest and the highest fortification level, respectively.

Analytical performance of the SLE‐DLLME with GC‐MS developed method for grape analysis provided comparable sensitivity values as regards LODs and LOQs than other previously proposed methods based on HS‐SPME [13, 14] with the advantage of applying the simple, rapid, and economic DLLME preconcentration approach if compared with the more expensive SPME fibers which require higher extraction times.

### 3.4 Analysis of grapes

The optimized method was applied to the analysis of four samples of grapes, differentiating between free and bound fractions of the studied compounds. The results obtained are shown in Table 5.

**TABLE 5:** Aroma compounds content^a^ (ng/g) in grapes

**Compound**	**Fraction**	**Grape 1**	**Grape 2**	**Grape 3**	**Grape 4**
Benzyl alcohol	Free	ND	ND	ND	NQ
	Bound	155 ± 14	198 ± 15	223 ± 13	NQ
Linalool oxide II	Free	NQ	ND	ND	ND
	Bound	52 ± 1	ND	ND	ND
Linalool oxide I	Free	ND	NQ	NQ	NQ
	Bound	127 ± 3	464 ± 18	ND	ND
Linalool	Free	359 ± 1	370 ± 3	441 ± 9	470 ± 4
	Bound	ND	ND	ND	ND
2‐Phenylethanol	Free	ND	ND	ND	ND
	Bound	105 ± 7	NQ	NQ	NQ

^a^ Mean value ± standard deviation (*n* = 3). ND, not detected. NQ, not quantified.

Only five of the compounds studied were detected in the studied samples: benzyl alcohol, 2‐phenylethanol, linalool, and linalool oxide I and II. Except for linalool, the detected aroma compounds showed much higher concentrations for the glycosylated than the corresponding free forms, in accordance with previously published results [5, 9]. For all the samples, linalool was the aroma compound detected in higher concentration (359–470 ng/g) in all cases under the free form. The contents of aroma compounds in the free phase were around 359, 370, 441, and 470 ng/g for samples 1–4, respectively, corresponding almost totally to linalool, as levels lower than the corresponding LOQs were found for benzyl alcohol and linalool oxide I and II in some of the grapes. Total concentrations for glycosylated forms were 439, 662, and 223 ng/g for samples 1, 2, and 3, respectively, while for sample 4 no quantifiable levels of linked fractions were found.

**FIGURE 2:**
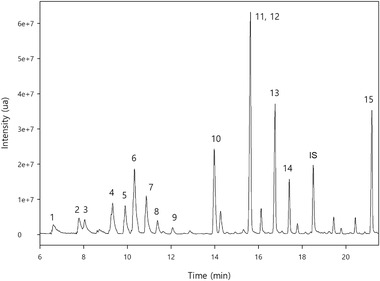
Total ion chromatogram (TIC) obtained for grape sample 1 fortified at 200 ng/g analyzed by the proposed solid‐liquid extraction–dispersive liquid‐liquid microextraction (SLE‐DLLME) with GC‐MS method. The peaks correspond to: 1, myrcene; 2, limonene; 3, benzyl alcohol; 4, linalool oxide II; 5, linalool oxide I; 6, linalool; 7, rose oxide II; 8, 2‐phenylethanol; 9, rose oxide I; 10, α‐terpineol; 11, citronellol; 12, nerol; 13, geraniol; 14, citral; 15, eugenol and IS, internal standard

Figure 2 shows the total ion chromatogram obtained for grape sample 1 fortified at 200 ng/g (80 ng/g for the IS) analyzed using the developed SLE‐DLLME and GC‐MS method under selected ion monitoring mode.

The identification of the analytes was carried out by contrasting the retention time of the compounds, identifying the *m/z* values for the target (T) and qualifier ions, as well as qualifier‐to‐target ratios (Q/T) of the peaks in standard solutions, samples, and fortified samples.

## 4 CONCLUDING REMARKS

The DLLME technique has demonstrated its capacity for the pre‐concentration of fifteen aroma compounds related to the organoleptic characteristics of grapes, in a simple, fast, and with a minimum organic solvent consumption procedure. The combination of DLLME with GC‐MS has provided analyses with a high degree of sensitivity and selectivity. Enzymatic hydrolysis applied prior to the preconcentration step allowed the differentiation of free and glycosylated forms of the studied compounds. The proposed SLE‐DLLME sample treatment simplified the sample matrix related to previously published methodologies, being the first time that this preconcentration/cleaning strategy is applied for the determination of aroma compounds in grapes. In addition, the simplicity of the organic extracts obtained allowed the quantification of the grape samples to be carried out by means of aqueous standards.

## CONFLICT OF INTEREST

The authors declare that they have no conflict of interest.

## Supporting information


**Figure S1**. Chemical structures for monitored aroma compounds
**Figure S2**. Effect of three factors on DLLME extraction efficiency of aroma compounds
**Table S1**. Recovery studies

Supplementary information

Supplementary information

## Data Availability

The data that support the findings of this study are available from the corresponding author upon reasonable request.
