# Supplementary material for: Free and glycosylated aroma compounds in grapes monitored by solid‐liquid extraction and dispersive liquid‐liquid microextraction combined with gas chromatography‐mass spectrometry
Source: J Sep Sci. 2022 Jun 25;45(15):2996–3004. doi: 10.1002/jssc.202200181 (PMC9546298; doi:10.1002/jssc.202200181)
Supplement: Supplementary file 1 — Figure S1. Chemical structures for monitored aroma compounds Figure S2. Effect of three factors on DLLME extraction efficiency of aroma compounds Table S1. Recovery studies [file JSSC-45-2996-s001.docx]

**Free and glycosylated aromatic compounds in grape monitored by dispersive liquid-liquid microextraction combined with gas chromatography and mass spectrometry**

Ainhoa Oller-Ruiz^1^, Pilar Viñas^1^, Manuel Hernández-Córdoba^1^, José Fenoll^2^, Isabel Garrido^2^, Natalia Campillo^1^*

*^1^Department of Analytical Chemistry, Faculty of Chemistry, Regional Campus of International Excellence “Campus Mare Nostrum”, University of Murcia, E-30100 Murcia, Spain*

*^2^Sustainability and Quality Group of Fruit and Vegetable Products. Murcia Institute of Agri-Food Research and Development. C/ Mayor s/n. La Alberca, 30150, Murcia, Spain*

*Corresponding author:

# Dr. Natalia Campillo

Department of Analytical Chemistry

Faculty of Chemistry

University of Murcia

E-30071 Murcia (SPAIN)

Tel.: +34 868887320

FAX: +34 868887682

e-mail: [pilarvi@um.es](mailto:pilarvi@um.es)

<http://www.um.es/aim>

**Fig. S1.** Chemical structures for monitored aroma compounds.

**Fig. S2.** Effect of three factors on DLLME extraction efficiency of aroma compounds.

| **Table 1S.** Recovery studies | | | |
| --- | --- | --- | --- |
| Compound | Fortification  level (ng g^-1^) | Grape sample 1 | Grape sample 4 |
| Myrcene | 150 | 82 | 96 |
|  | 250 | 97 | 98 |
| Limonene | 150 | 84 | 84 |
|  | 250 | 114 | 100 |
| Benzyl alcohol | 150 | 114 | 83 |
|  | 250 | 115 | 101 |
| Linalool oxide II | 150 | 92 | 87 |
|  | 250 | 107 | 105 |
| Linalool oxide I | 150 | 84 | 93 |
|  | 250 | 104 | 108 |
| Linalool | 150 | 114 | 77 |
|  | 250 | 102 | 99 |
| Rose oxide II | 150 | 89 | 88 |
|  | 250 | 105 | 104 |
| 2-Phenylethanol | 150 | 110 | 115 |
|  | 250 | 115 | 112 |
| Rose oxide I | 150 | 85 | 93 |
|  | 250 | 102 | 103 |
| α-Terpineol | 150 | 81 | 92 |
|  | 250 | 105 | 103 |
| Citronellol | 150 | 94 | 90 |
|  | 250 | 104 | 99 |
| Nerol | 150 | 95 | 86 |
|  | 250 | 104 | 101 |
| Geraniol | 150 | 113 | 80 |
|  | 250 | 109 | 99 |
| Citral | 150 | 97 | 90 |
|  | 250 | 111 | 101 |
| Eugenol | 150 | 91 | 83 |
|  | 250 | 106 | 99 |
