# Supplementary figures and images for: Free and glycosylated aroma compounds in grapes monitored by solid‐liquid extraction and dispersive liquid‐liquid microextraction combined with gas chromatography‐mass spectrometry
Source: J Sep Sci. 2022 Jun 25;45(15):2996–3004. doi: 10.1002/jssc.202200181 (PMC9546298; doi:10.1002/jssc.202200181)

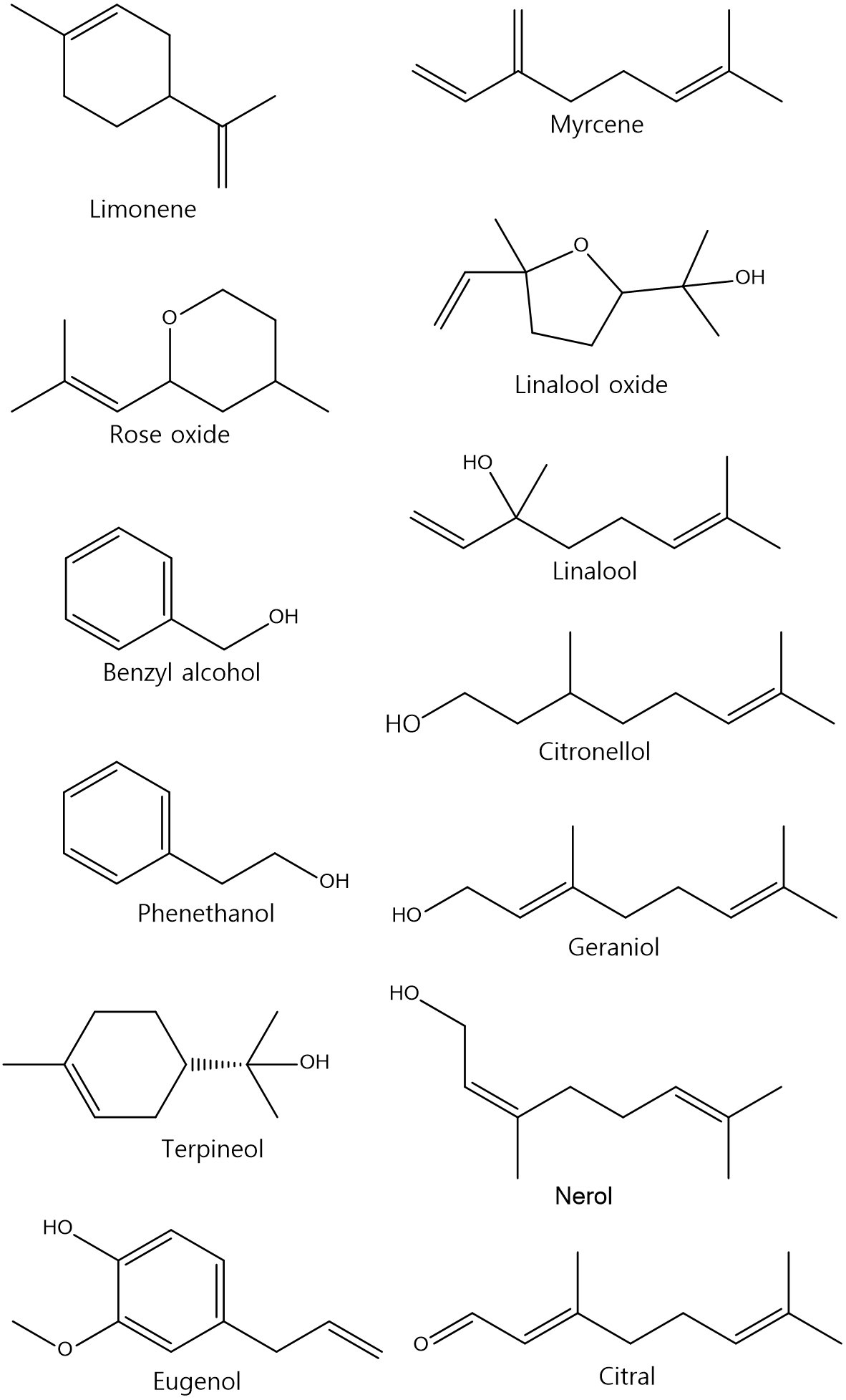

Supplement: Supplementary file 2 — Supplementary information [file JSSC-45-2996-s003.tif]
